# Overexpression and simple purification of the *Thermotoga maritima *6-phosphogluconate dehydrogenase in *Escherichia coli *and its application for NADPH regeneration

**DOI:** 10.1186/1475-2859-8-30

**Published:** 2009-06-04

**Authors:** Yiran Wang, Y-H Percival Zhang

**Affiliations:** 1 Biological Systems Engineering Department, 210-A Seitz Hall, Virginia Polytechnic Institute and State University, Blacksburg, Virgina 24061, USA; 2 Institute for Critical Technology and Applied Sciences (ICTAS) Virginia Polytechnic Institute and State University, Blacksburg, Virginia 24061, USA; 3 DOE BioEnergy Science Center (BESC), Oak Ridge, Tennessee 37831, USA

## Abstract

**Background:**

Thermostable enzymes from thermophilic microorganisms are playing more and more important roles in molecular biology R&D and industrial applications. However, over-production of recombinant soluble proteins from thermophilic microorganisms in mesophilic hosts (e.g. *E. coli*) remains challenging sometimes.

**Results:**

An open reading frame TM0438 from a hyperthermophilic bacterium *Thermotoga maritima *putatively encoding 6-phosphogluconate dehydrogenase (6PGDH) was cloned and expressed in *E. coli*. The purified protein was confirmed to have 6PGDH activity with a molecular mass of 53 kDa. The *k*_*cat *_of this enzyme was 325 s^-1 ^and the *K*_*m *_values for 6-phosphogluconate, NADP^+^, and NAD^+ ^were 11, 10 and 380 μM, respectively, at 80°C. This enzyme had half-life times of 48 and 140 h at 90 and 80°C, respectively. Through numerous approaches including expression vectors, hosts, cultivation conditions, inducers, and codon-optimization of the *6pgdh *gene, the soluble 6PGDH expression levels were enhanced to ~250 mg per liter of culture by more than 500-fold. The recombinant 6PGDH accounted for >30% of total *E. coli *cellular proteins when lactose was used as a low-cost inducer. In addition, this enzyme coupled with glucose-6-phosphate dehydrogenase for the first time was demonstrated to generate two moles of NADPH per mole of glucose-6-phosphate.

**Conclusion:**

We have achieved a more than 500-fold improvement in the expression of soluble *T. maritima *6PGDH in *E. coli*, characterized its basic biochemical properties, and demonstrated its applicability for NADPH regeneration by a new enzyme cocktail. The methodology for over-expression and simple purification of this thermostable protein would be useful for the production of other thermostable proteins in *E. coli*.

## Background

Enzyme-based biocatalysis has become an attractive alternative to chemical catalysis because of its higher reaction selectivity and more modest reaction conditions [1,2]. But most enzymes are not suitable for industrial applications due to their relatively poor stability and biocatalyst re-use. The former can be addressed by protein engineering [3-5], enzyme immobilization [6,7], utilization of stable enzymes from extremophilic microorganisms [8,9], or their combinations [10-12]. The latter can be solved through enzyme immobilization [6,7]. For example, immobilized thermostable glucose isomerase has been used in the food industry to convert glucose to fructose at ~60°C for several months before its deactivation [10].

Discovery and utilization of thermoenzyme from (hyper)thermophilic microorganisms is of great interest for numerous applications. *Thermotoga maritima *is an anaerobic, rod-shaped eubacterium, originally isolated from geothermally heated marine sediment at Valcano, Italy. It has an optimum growth temperature of ~80°C [13]. *T. maritima *is regarded as an invaluable source of intrinsically thermostable enzymes [14,15]. The open reading frame (ORF) TM0438 was annotated to be a 6-phosphogluconate dehydrogenase (6PGDH, E.C.1.1.1.44) [16], but its biochemical function has not yet been confirmed.

*E. coli *is a common prokaryotic microorganism for genetic manipulation and for the production of recombinant proteins because of its fast cell growth in inexpensive media, rapid accumulation of cellular mass, amenability to high cell-density fermentation, simple scale-up, and relatively simple protein purification [17]. But *E. coli *often produces recombinant protein in the form of insoluble, inactive inclusion bodies. It is estimated that less than 20% of the ORFs in other genomes are likely to be expressed as soluble active proteins in *E. coli *[18]. A number of approaches have been explored to improve the expression of soluble recombinant proteins in *E. coli*. With regard to the expression vector, the heterologous protein could be fused with a protein-folding partner (e.g. thioredoxin, cellulose-binding module) [19-21] or with a secretory protein fragment (e.g. outer-membrane protein A) that aids protein folding in a less-reducing periplasmic environment [17]. Expression hosts can be chosen according to different approaches, such as (i) mitigating codon bias in a host containing a second plasmid expressing the *E. coli *rare tRNA genes [22], (ii) enhancing protein folding in a host co-expressing folding modulators, such as chaperons [17,23], (iii) decreasing formation of disulfide bond in some special host's cytoplasm [24], and (iv) repressing basal expression of a toxic protein in a host with a repressor [25]. In addition, cultivation conditions, such as expression temperature, medium composition, timing of induction, inducer concentration, and inducer type, can be optimized for over-expression of a soluble protein [26,27]. Recently, synthetic codon-optimized genes have been more adapted for heterologous protein expression [28-30]. But over-expression of soluble heterologous proteins, especially for hyperthermophilic ones, in *E. coli *still remains on a trial-and-test stage [22,31,32].

6-phosphogluconate dehydrogenase is responsible for converting 6-phosphogluconate to ribulose-5-phosphate and CO_2_, along with one NADPH generation from NADP^+ ^[33-35]. Thermostable 6PGDH has some potential applications, such as generation of high-yield hydrogen from sugars [36,37] and biosynthesis of chiral alcohols. For the production of a third generation biofuel – hydrogen, utilization of thermostable enzymes would increase production rates and stabilize the enzyme at elevated temperature [12,36,37]. Biosynthesis of chiral alcohols mediated by enzymes requires low-cost regeneration of NAD(P)H [38-41]. Glucose-6-phosphate dehydrogenase (G6PDH) has been applied to generate one NADPH per glucose-6-phospahte [38,42,43]. The combination of G6PDH and 6PGDH may double NADPH yield from costly glucose-6-phosphate, but no such study has been reported.

In this study, we cloned the ORF TM0438 encoding a putative *T. maritima *6PGDH and purified and characterized the enzyme. Using different approaches, we increased its expression levels in *E. coli *from hardly-detectable to more than 250 mg per liter of culture. Also, we demonstrated that two moles of NADPH per mole of glucose-6-phosphate were generated by using an enzyme cocktail containing G6PDH and 6PGDH.

## Results

### Expression of wild-type *6pgdh *gene

**Figure 1:**
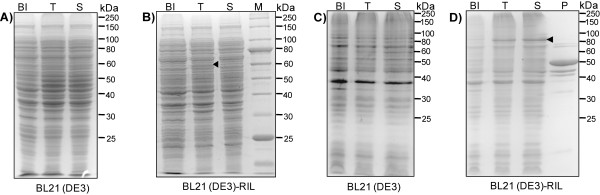
**SDS-PAGE analysis of the expression of the *wt6pgdh *gene**. (A) pET-trx-wt6pgdh in *E. coli *BL21(DE3), (B) pET-trx-wt6pgdh in *E. coli *BL21(DE3)-RIL, (C) pET-ci-wt6pgdh in *E. coli *BL21(DE3) and (D) pET-ci-wt6pgdh in *E. coli *BL21(DE3)-RIL. BI, before induction; T, total cellular proteins; S, soluble proteins; P, purified protein. Cells were grown at 37°C until A_600 _of 0.6. The induction condition was 20 μM IPTG at 20°C for 6 h.

The expression plasmid pET-trx-wt6pgdh was constructed based on a pET102-TOPO plasmid. This construct encodes a fusion protein with a N-terminal thioredoxin, which is well-known to enhance expression of soluble heterologous proteins in *E. coli *[21]. The *E. coli *BL21(DE3) harboring plasmid pET-trx-wt6pgdh did not produce a detectable protein band corresponding to the size of the fusion protein under various experimental conditions (expression temperatures from 15, 20, 30 to 37°C and IPTG concentrations from 20, 100 to 500 μM). Figure 1A shows a typical result of the cell lysate of *E. coli *BL21(DE3) induced by 500 μM IPTG at 20°C.

Since the codon usage of *E. coli *BL21(DE3) is drastically different from that of *T. maritima, E. coli *BL21(DE3)-RIL that carries a ColE1-compatible vector encoding extra copies of tRNA genes of *argU*, *ileY*, and *leuW *was used to over-express soluble 6PGDH. *E. coli *BL21(DE3)-RIL/pET-trx-wt6pgdh produced a weak protein band corresponding to the size of the fusion protein under the conditions (500 μM IPTG and 20°C), but a majority of the fusion protein was insoluble (Figure 1B). Decreasing IPTG concentration to 20 μM did not improve the expression of soluble 6PGDH (data not shown), although lowering IPTG concentrations often improved the expression of soluble protein [27].

Because cellulose-binding module (CBM) tags have been reported to enhance heterologous protein expression and folding in *E. coli *[20,44], we attempted to express 6PGDH by replacing the thioredoxin tag with a *Clostridium thermocellum *family 3 CBM tag linked with an intein. Regardless of IPTG concentrations (20 or 500 μM IPTG), the *E. coli *BL21(DE3)/pET-trx-wt6pgdh did not produce any obvious soluble or insoluble protein bands corresponding to the right size (Figure 1C, 20 μM IPTG). But BL21(DE3)-RIL bearing the expression plasmid pET-trx-wt6pgdh produced some soluble 6PGDH (Figure 1D, 20 μM IPTG, 20°C). These results suggested that *E. coli *BL21(DE3)-RIL enhanced expression of the *wt6pgdh *and the CBM tag helped expression of soluble 6PGDH more efficiently than did thioredoxin.

Through affinity adsorption of CBM-tagged 6PGDH on regenerated amorphous cellulose followed by intein self-cleavage [44], approximately eight mg of 6PGDH was purified per liter of the culture. But the purified 6PGDH was composed of several small-size proteins (Lane P, Figure 1D), suggesting possible proteolysis or incomplete translation. The first cause was eliminated because addition of a protease inhibitor phenylmethanesulfonyl fluoride during cell disruption and protein purification did not change the composition of the small-size proteins (data not shown).

### Codon analysis and optimization

**Figure 2:**
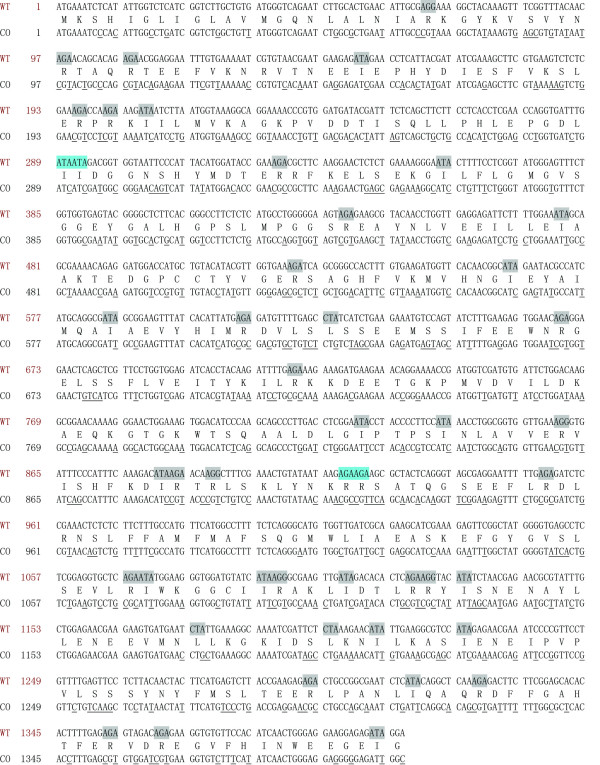
**The wild-type *6pgdh *sequence, the deduced amino acid sequence, and the codon-optimized *6pgdh *sequence**. WT, *wt6pgdh*; CO, *co6pgdh*. The highlighted codons among the *wt6pgdh *gene are the rare codons in *E. coli*. The codon clusters in blue are the rare codons sites for site-directed mutagenesis. The underlined nucleotides in *co6pgdh *are the changed ones corresponding to *wt6pgdh*.

Figure 2 shows the *wt6pgdh *DNA sequence, the deduced amino acids, and the codon-optimized DNA sequence (*co6pgdh*). The *wt6pgdh *gene contains 47 rare *E. coli *codons, accounting for ~10% of the entire sequence. They are 20 AGA, 5 AGG, 19 AUA, and 3 CUA. The AGA(Arg) and AUA(Ile) codon frequencies in the *wt6pgdh *gene are 4.3% and 4.0%, but are only 0.24% and 0.5% in *E. coli *, respectively. Moreover, two rare codons formed clusters AUA(Ile97)-AUA(Ile98) and AGA(Arg306)-AGA(Arg307). Following site-directed mutagenesis to remove these two rare-codon clusters, there were no noticeable changes in the SDS-PAGE patterns of the purified 6PGDHs before and after site-directed mutagenesis (data not shown). Therefore, the entire *wt6pgdh *DNA sequence was optimized to remove all 47 rare codons by using frequently-used *E. coli *codons, based on several rules: (i) keeping the GC ratio around 50%; (ii) avoiding *cis*-acting DNA sequences (internal TATA-boxes, chi-sites, and ribosomal entry sites; AT-rich or GC-rich sequence stretches; repeat sequences; and RNA secondary structures); (iii) precluding cutting sites of frequently-used restriction enzymes, and (iv) adding two stop TAATAA to ensure efficient termination of translation, and (v) using a strong terminator in the expression vector for enhancing mRNA stability. The overall GC content for the codon-optimized 6PGDH was 49% (Figure 2).

### Expression of the codon-optimized *6pgdh *gene

**Figure 3:**
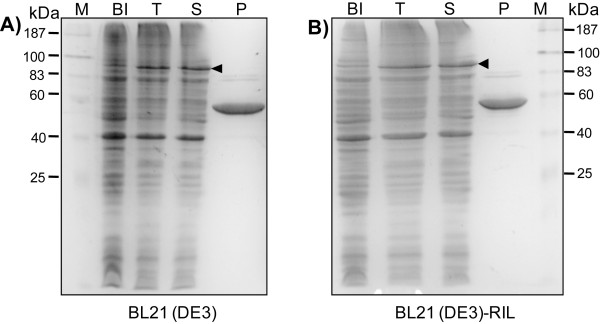
**SDS-PAGE analysis of the expression and purification of *T. maritima *6PGDH from plasmid pET-ci-co6pgdh in the *E. coli *BL21(DE3) (A) and *E. coli *BL21(DE3)-RIL (B)**. M, protein marker; BI, before induction; T, total cellular proteins; S, soluble proteins; P, purified protein. Cells were grown at 37°C until A_600 _of 0.6. The induction condition was 20 μM IPTG at 20°C for 6 h.

Plasmid pET-ci-co6pgdh encoding the fusion protein CBM-intein-6PGDH was expressed in *E. coli *BL21(DE3) and BL21(DE3)-RIL, separately. Figure 3 shows that both hosts produced soluble target proteins at similar levels, suggesting that *E. coli *BL21(DE3)-RIL was not necessary for expression of the codon-optimized gene. After purification, no small-size protein fragments accompanied with the purified 6PGDH were observed (Figure 3), suggesting that the rare codons mainly caused the incomplete translation (Figure 1D). Approximately 15–17 mg of 6PGDH was purified per liter of culture for both hosts.

### Basic biochemical characterization of *T. maritima *6PGDH

**Figure 4:**
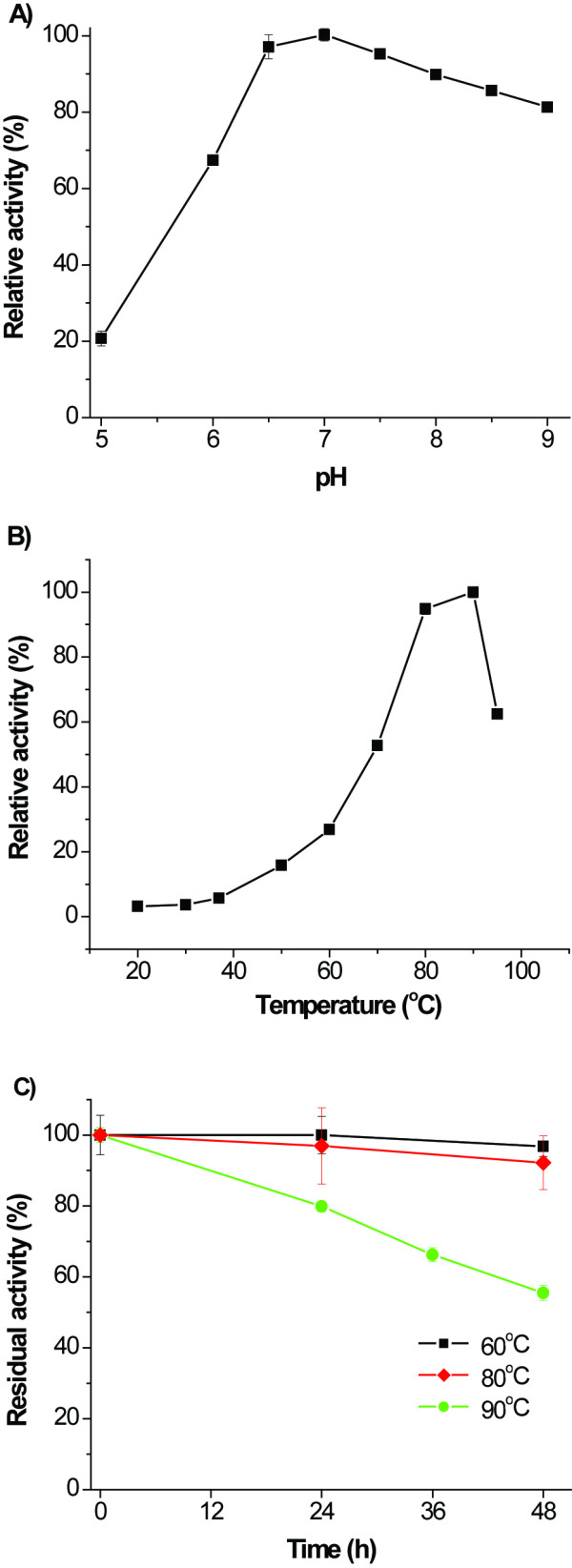
**The reaction conditions of pH (A) and temperature (B) as well as thermostability (C) at an enzyme concentration of 0.24 mg/mL**.

The cleaved *T. maritima *6PGDH through RAC adsorption and intein cleavage was purified and characterized. The pH effects on 6PGDH activity were studied in 50 mM citric acid/sodium citrate buffers (pH 5.0 and 6.0), a Bis-Tris (pH 6.5), Tris-HCl buffers (pH 7.0, 7.5 8.0, 8.5, and 9.0), and a Hepes buffer (pH 7.5). The optimum pH was found to be around pH 7.0 (Figure 4A). About 70% of 6PGDH activities remained at pH 6.0 and 9.0. The enzyme had the similar activities in the Hepes and Tris buffers (pH 7.5).

The effects of temperature on *T. maritima *6PGDH activities were measured from 20 to 95°C (Figure 4B). The optimum temperature was around 90°C. The 6PGDH had an approximately 90% of its maximum activity at 80°C, but retained only ~2 and ~20% of its maximum activity at 30 and 60°C, respectively. The activation energy was 51.4 kJ/mol at a temperature range of 20–80°C, based on the Arrhenius plot. The 6PGDH was highly thermostable (Figure 4C) in a 50 mM Hepes buffer (pH 6.8) containing 500 mM NaCl, 1 mM EDTA, and 5 mM β-mercaptoethanol. It retained more than 90% activity for 48 h at 60 and 80°C and retained ~50% enzymatic activity after 48 h at 90°C. This enzyme had half-life times of 48 and 140 h at 90 and 80°C, respectively.

The kinetics properties of *T. maritima *6PGDH followed the Michaelis-Menten equation. The apparent *K*_*m *_values were 11, 10, and 380 μM on 6-phosphogluconate, NADP^+^, and NAD^+^, respectively and *k*_*cat *_value was 325 s^-1^. Clearly, this enzyme preferred NADP^+ ^to NAD^+ ^as an electron acceptor.

### Overexpression of *co6pgdh *and simple purification

**Figure 5:**
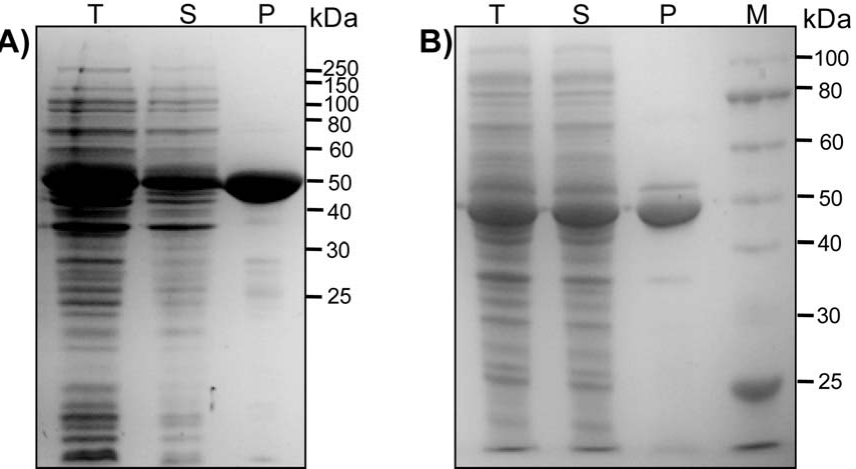
**SDS-PAGE analysis of the expression and purification of *T. maritima *6PGDH from the *E. coli *BL21(DE3)/pET-co6pgdh-his (A) and *E. coli *BL21(DE3)/pET-co6pgdh (B)**. Cells were grown at 37°C until A_600 _of 0.6. The induction condition was 500 μM IPTG at 37°C for 4 h.

Codon analysis indicated that the *C. thermocellum *CBM and the *Synechocystis *intein contained 21 rare codons. In addition, self-cleavage of intein during protein expression and cell disruption may result in some loss of the desired protein even at a decreased cultivation temperature [44]. Expression vector pET-co6pgdh-his was constructed to express a *co6pgdh *gene with a C-terminal His-tag. Figure 5A shows the SDS-PAGE analysis of 6PGDH expression. More than 200 mg of 6PGDH-His was purified per liter of culture. But the specific enzymatic activity of the 6PGDH-His was approximately 80% of that of 6PGDH without the His tag.

Since *T. maritima *6PGDH was extremely thermostable (Figure 4C), heat precipitation was chosen for simplifying protein purification. Plasmid pET-co6gpdh was constructed for expressing the *co6gpdh *gene without a His-tag. The cell lysate was treated at 90 or 100°C with time lengths ranging from 15 minute to 6 hours. The highest 6PGDH yield was obtained under heat treatment conditions (90°C for 30 min), where a 6PGDH purity was around 85%, as judged by SDS-PAGE analysis (Figure 5B). The overall recovery yield was 90% according to 6PGDH activity. Approximately 190 mg of 6PGDH was obtained from the cells harvested after 4-hour induction at 37°C.

**Figure 6:**
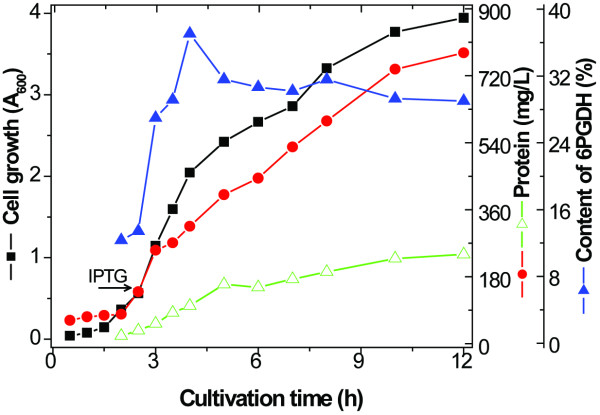
**Cultivation profiles of cell growth, total cellular protein, and *T. maritima *6PGDH content by *E. coli *BL21(DE3)/pET-co6pgdh in the LB medium at 37°C**.

Figure 6 shows the fermentation profiles of *E. coli *BL21(DE3)/pET21-co6pgdh in 200 mL of LB medium in a 1-L Erlenmeyer flask at a constant cultivation temperature of 37°C. When the A_600 _reached 0.6, 500 μM of IPTG was added. The highest A_600 _was 3.9, and the total cell protein was 790 mg per liter at hour 12 (9.5 hours after IPTG induction). The 6PGDH content rose from ~10% before induction to 38% at hour 4.5 and then decreased to levels of ~30% for the remaining cultivation period. Up to 230 mg of 6PGDH was produced from one liter of the LB-grown culture.

To decrease the inducer cost, different concentrations of IPTG (20, 100 or 500 μM) as well as lactose (100 μM) were further investigated. Nearly all cells had similar growth patterns except that 500 μM IPTG slightly inhibited cell growth during the first six-hour induction. After cell lysis and heat precipitation, approximately 194, 224, 208, and 250 mg of the 6PGDH protein were obtained under the conditions of 20, 100, and 500 μM IPTG, and 100 μM lactose, respectively. The largest amount of 6PGDH was obtained when lactose was used as the inducer, suggesting that low-cost lactose was an effective inducer and worked as a supplementary carbon source for protein synthesis.

### NADPH regeneration

**Figure 7:**
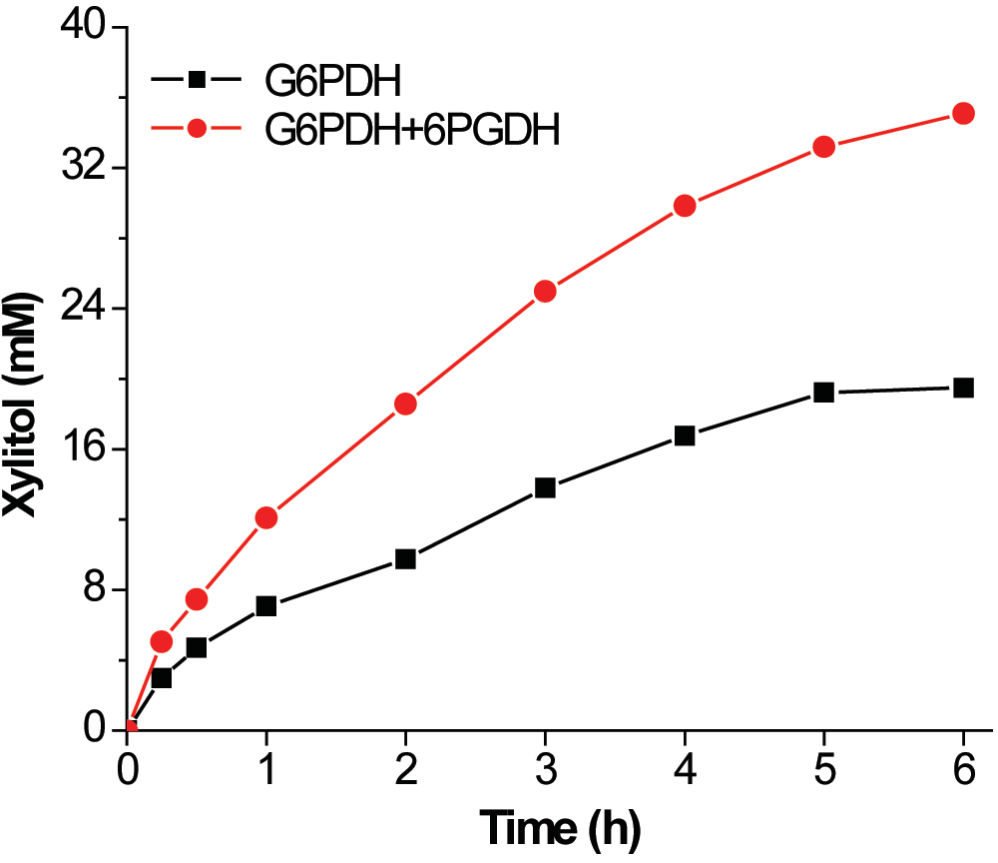
**Profile of xylitol synthesis coupled with NADPH regeneration reaction from glucose-6-phosphate mediated by G6PDH alone or coupled with 6PGDH**.

Coupling of 6PGDH and G6PDH was believed to generate one more mole of NADPH per mole of glucose-6-phosphate relative to G6PDH alone. Xylitol can be produced from xylose and NADPH mediated by xylose reductase [45,46]. Figure 7 shows kinetics of xylitol synthesis with glucose-6-phosphate as the NADPH regeneration substrate and G6PDH in the presence and absence of 6PGDH. In the case of three enzyme cocktails (G6PDH, 6PGDH, and xylose reductase), 35.5 mM xylitol was produced at hour six, nearly twice that of the two-enzyme system (19.5 mM, G6PDH and xylose reductase). The yields of xylitol synthesized were 175% and 97%, respectively, relative to glucose-6-phosphate consumed for the reactions mediated by the three enzymes and by the two enzymes.

## Discussion

**Figure 8:**
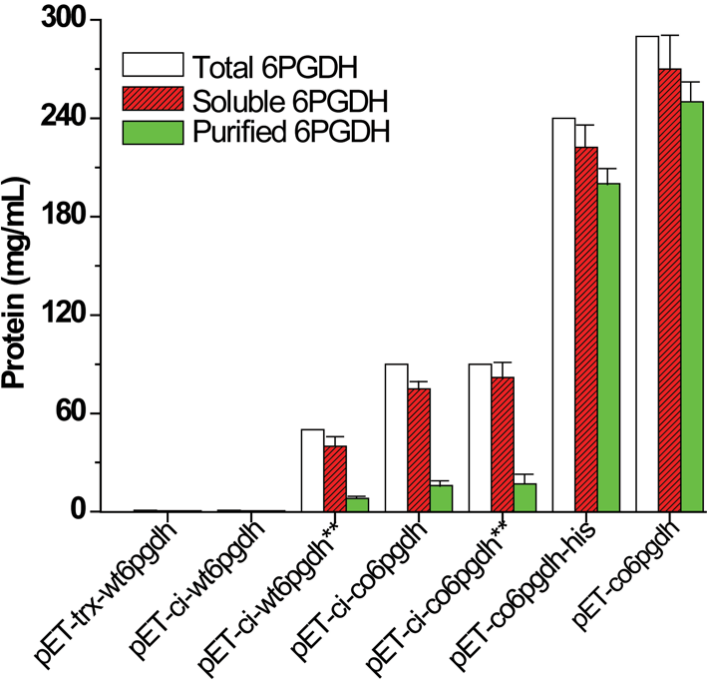
**Production and purification of *T. maritima *6PGDH**. The 6PGDH amounts were calculated based on their activity relative to the purified 6PGDH specific activity. The amount of total *T. maritima *6PGDH proteins produced by *E. coli *were estimated from the band intensities on the SDS-PAGE gels relative to those contained in the soluble fraction. Expression host was *E. coli *BL21(DE3) except where ** denoted *E. coli *BL21(DE3)-RIL. The data from left to right were obtained from Figure 1A, Figure 1C, Figure 1D, Figure 3A, Figure 3B, Figure 5A, and the expression by the *E. coli *BL21(DE3)/pET-co6pgdh (induced by 100 μM lactose at 37°C for 10 h).

The ORF TM0438 was confirmed to encode a hyperthermophilic 6PGDH. Through different approaches (expression vectors, hosts, cultivation conditions, inducer type, and gene sequence), we increased expression levels of the soluble *T. maritima *6PGDH from nearly-undetectable to more than 250 mg per liter of the LB-grown culture, as summarized in Figure 8. Several lessons were learned from this study. (A) The codon bias between the rare codon-rich *wt6pgdh *gene and the expression host *E. coli *was the largest cause for its low expression. It can be addressed (i) mainly by a codon-optimized synthetic gene (Figure 5) or (ii) partially by using *E. coli *BL21(DE3)-RIL (Figure 1). (B) The *C. thermocellum *CBM tag enhanced *T. maritima *6PGDH folding more efficiently than did thioredoxin (Figure 1D*vs *Figure 1B). (C) Decreasing cultivation temperature and/or inducer concentration possibly decreased formation of inclusion body. But it was not necessary for the expression of the codon-optimized *6pgdh *gene (Figure 5). (D) The 6PGDH was induced by a low level of IPTG or lactose. Low-cost lactose was highly recommended because it was used as the carbon source for supporting cell growth and protein synthesis (low cost plus high protein yield). (E) More than 30% of the *E. coli *cellular protein was soluble *T. maritima *6PGDH after numerous approaches. (F) Heat precipitation was effective to obtain relatively pure thermostable enzymes (Figure 5B), suggesting that feasibility of ultra-low-cost mass production of this thermostable enzyme.

Few studies have been conducted pertaining to cloning and characterization of 6-phosphogluconate dehydrogenase [47,48]. The *T. maritima *6PGDH is the most thermostable 6PGDH characterized so far, with half-life times of 48 h and ~140 h at 90°C and 80°C, respectively. This enzyme was far more thermostable than the *Bacillus stearothermophilus *6PGDH with a half-life time of about 15 min at 70°C [49]. The hyper-thermostability of *T. maritima *6PGDH makes it possible to simplify its purification by heat precipitation, different from most protein purification technologies, such as chromatography or adsorption/desorption [19,44]. Heat precipitation is becoming a popular protein purification protocol for hyper-thermostable proteins [50,51].

NAD(P)H enzymatic regeneration can be conducted by combining a number of enzyme/substrates, for example, glucose dehydrogenase [52], formate dehydrogenase/formate [53], phosphate dehydrogenase/phosphite [46], G6PDH/glucose-6-phosphate [42,43,54], and so on. Starting from G6PDH and glucose-6-phosphate, the addition of 6PGDH doubled NADPH yield to two NADPH per glucose-6-phosphate, resulting in 1.8-fold xylitol production as compared to G6PDH alone (Figure 7). This new enzyme cocktail would make the NADPH regeneration from glucose-6-phosphate more economically feasible.

High expression levels of *T. maritima *6PGDH in *E. coli *(> 30% of total cellular protein), simple purification by heat precipitation and its hyper-thermostability suggest great potential for decreasing protein costs associated with its production, separation, and use. Although costly LB medium was used for production of 6PGDH in flasks on a laboratory scale, the production cost of 6PGDH is anticipated to decrease greatly by using low-cost lean medium plus high-cell-density fermentation in bioreactors. This highly-thermostable 6PGDH would be invaluable for high-yield generation of hydrogen from polysaccharides and water mediated by cell-free synthetic pathway biotransformation (SyPaB) [12,36,37].

## Conclusion

In conclusion, we over-expressed more than 250 mg of *T. maritima *6PGDH per liter of culture through numerous approaches, characterized its basic biochemical properties, and demonstrated its applicability for high-yield NADPH regeneration. This hyper-thermostable 6PGDH was easily purified by heat precipitation. The methodology for over-expression and simple purification of this thermostable protein would be useful for the production of other thermostable proteins in *E. coli*.

## Methods

### Chemicals, plasmids, and strains

All chemicals were of reagent grade, purchased from Sigma (St. Louis, MO) and Fisher Scientific (Pittsburgh, PA), unless otherwise noted. Regenerated amorphous cellulose (RAC) was prepared through cellulose dissolution by ice-cooled concentrated phosphoric acid followed by regeneration in water [55]. *Pfx*50 DNA polymerase, Champion™ pET102 Directional TOPO^® ^Expression Kit with *E. coli *BL21 Star™ (DE3), and Ni-NTA agarose were purchased from Invitrogen (Carlsbad, CA). The *T. maritima *genomic DNA was purchased from the American Type Culture Collection (Manassas, VA). The strains, plasmids, and oligonucleotides used in this study are listed in Table 1.

**Table 1:** The strains, plasmids, and oligonucleotides used in this study

Description	Contents	Reference/sources
Strain		
*E. coli *Bl21^star^(DE3)	B F^- ^*ompT hsdS*_B_(r_B_^-^m_B_^-^) *gal dcm rne131 *(DE3)	Invitrogen
*E. coli *Bl21(DE3)-RIL	B F^- ^*ompT hsdS*(r_B_^-^m_B_^-^) *dcm *Tet^r ^*gal *λ(DE3) *endA *Hte [*argU ileY leuW *Cam^r^]	Stratagene
Plasmid		
pCIP	Amp^r^, T7 promoter, lacO, ColE1 ori, parental DNA, replacing *pgm *gene with *wt6pgdh*, *co6pgdh *or *g6pdh*	[44]
pET21a		Epoch Biolabs
pET-trx-wt6pgdh	Amp^r^, T7 promoter, lacO, ColE1 ori, Trx-wt6pgdh	This study
pET-ci-wt6pgdh	*wt6pgdh *gene subcloned into pCIP	This study
pET-ci-co6pgdh	*co6pgdh *gene subcloned into pCIP	This study
pET-co6pgdh-his	*co6pgdh *gene with C-terminal (His)_6 _cloned into pET21a	
pET-co6pgdh	Removed C-terminal (His)_6 _from pET-co6pgdh-his	This study
pET-ci-g6pdhp	Expression of *T. maritima g6pdh*	This study
pET26b-xr	Expression of *N. crassa *xylose reductase	[57]
Primers*		Final plasmid
Trx-F	5'-caccatggtgaaatctcatattggtctcatcggtc-3'	pET-trx-wt6pgdh
Trx-R	5'-tcatcctatctctccttcctcccagttg-3'	
CI-wt-F	5'-ccagtctactcgaggtgaaatctcatattggtctcatcggtc-3'	pET-ci-wt6pgdh
CI-wt-R	5'-ccagtctagtcgaccctatctctccttcctcccag-3'	
CI-co-F	5'-ccagtcta ctcgagggctcttccatgaaatcccacattggcctgatc-3'	pET-ci-co6pgdh
CI-co-R	5'-ccagtctaggatcctcaagtcgagccaatctccccctcctccc-3'	
NH-F	5'-gaggagggggagattggctaacatcaccaccaccattaag-3'	pET-co6pgdh
NH-R	5'-cttaatggtggtggtgatgttagccaatctccccctcctc-3'	
G6P-F	5'-ccagtctactcgagggctcttcc atgaagtgcagtctgggattg-3'	pET-ci-g6pdh
G6P-R	5'-ccagtctagtcgacagttttctccattttctacc-3'	

* Underlined nucleotide sequences indicate restriction endonuclease sites.

### Construction of expression plasmids

Five expression plasmids were constructed for expressing wild-type *6pgdh *(*wt6pgdh*) and codon-optimized *6pgdh *(*co6pgdh*) genes under the control of T7 promoter (Table 2). Plasmid pET-trx-wt6pgdh encoding a fusion protein of thioredoxin (Trx) and 6PGDH was constructed by insertion of amplified *wt6pgdh *gene into pET102 Directional TOPO^® ^. The *wt6pgdh *DNA fragment was PCR amplified using primers Trx-F and Trx-R from the *T. maritima *genomic DNA. Plasmid pET-ci-wt6pgdh was constructed by replacing the *pgm *gene in the plasmid pCIP [44] by the *wt6pgdh *gene. The whole *6pgdh *DNA sequence was optimized based on the codon usage for *E. coli *B , yielding the *co6pgdh *DNA sequence. The *co6pgdh *DNA sequence with a C-terminal His-tag was synthesized by Epoch Biolabs (Sugar Land, TX) and cloned into pET21a *via *the restriction endonuclease sites *Nde1 *and *BamH1 *to obtain pET-co6pgdh-his. Plasmid pET-ci-co6pgdh was constructed similarly to pET-ci-wt6pgdh using the *co6pgdh *DNA sequence. Plasmid pET-co6pgdh encoding 6PGDH protein without a His-tag was constructed based on pET-co6pgdh-his by replacing the first codon (CAT) of the His-tag with a stop codon (TAA). The site-directed mutagenesis was conducted by using primers NH-F and NH-R following QuikChange™ Site-Directed Mutagenesis (Stratagene, La Jolla, CA).

**Table 2:** Expression plasmids for *T. maritima *6PGDH with or without the tags

Plasmid	Modular organization	Molecular mass
pET-trx-wt6pgdh	Trx-WT6PGDH	66, 634 Da
pET-ci-wt6pgdh	CBM-intein-WT6PGDH	91, 187 Da
pET-ci-co6pgdh	CBM-intein-CO6PGDH	91, 187 Da
pET-co6pgdh-his	CO6PGDH-(His)_6_	53, 975 Da
pET-co6pgdh	CO6PGDH	53, 152 Da

### Protein expression and purification

The *E. coli *strain harboring the expression plasmid was grown in a 200 mL LB medium in a 1-L flask with appropriate antibiotics at 37°C until the A_600 _reached ~0.6. After addition of the inducer (isopropyl β-D-1-thiogalactopyranoside – IPTG or lactose), the culture were grown at 37°C or a lower temperature (e.g. 20°C). For the CBM-tag proteins, they were purified through affinity binding on RAC followed by intein self-cleavage [44]. For the His-tag proteins, the cells were disrupted by sonicator in a 50 mM Tris-HCl (pH 7.5) containing 500 mM NaCl, 5 mM imidazol, and 20% (w/v) glycerol. After centrifugation at 8,000 g for 5 min, the supernatant was collected and purified by using affinity binding on Ni-NTA resin. For purification through heat precipitation, the soluble fraction of *E. coli *cell lysate was incubated at 90°C for 30 min in a 50 mM Tris-HCl (pH 7.5) buffer containing 100 mM NaCl and 20% (w/v) glycerol. After centrifugation, the supernatant contained relatively pure 6PGDH.

The *T. maritima *glucose-6-phosphate dehydrogenase (G6PDH) was produced by *E. coli *BL21(DE3)-RIL/pET-ci-g6pdh. The G6PDH was purified through affinity binding on RAC followed by intein self-cleavage [44] and characterized as described [56]. The *Neurospora crassa *xylose reductase was expressed and purified as described elsewhere [57].

### 6PGDH activity assays

*T. maritima *6PGDH activity was measured in a 50 mM Hepes buffer (pH 7.5) containing 2 mM 6-phosphogluconate, 1 mM NADP^+^, 5 mM Mg^2+^, 0.5 mM Mn^2+^, and 0.5 mg bovine serum albumin per mL at 80°C for 5 min. The reaction product NADPH was measured at 340 nm by DU^® ^800 UV/visible spectrophotometer (Beckman Coulter, Fullerton, CA). The enzyme unit was defined as one μmole of NADPH produced per min. For determining enzyme kinetic parameters, the *K*_*m *_of 6-phosphogluconate was measured in a 50 mM Hepes (pH 7.5) buffer containing 1 mM NADP^+^, 5 mM Mg^2+^, 0.5 mM Mn^2+^, along with 2.5 to 50 μM 6-phosphogluconate; the *K*_*m *_of NADP^+ ^was measured in the same buffer 2 mM 6-phosphogluconate with various concentrations of NADP^+ ^from 2.5 to 50 μM. The *K*_*m *_of NAD^+ ^was measured using 50 to 1000 μM NAD^+^.

#### Protein assays

Concentration of soluble protein was measured by the Bio-Rad Bradford protein kit with bovine serum albumin (BSA) as a standard protein. Total cellular protein was measured as described previously [58].

### Xylitol production with NADPH regeneration

Synthesis of xylitol from xylose mediated by xylose reductase was conducted in a 200 μL reaction volume in the presence of G6PDH or G6PDH/6PGDH at 25°C. The reaction mixture contained a 50 mM Hepes buffer (pH 7.5) with 50 mM xylose, 1 mg xylose reductase/mL, 2 mM NADP^+^, 20 mM glucose-6-phospahte, 0.16 mg *T. maritima *6PGDH/mL, 0.3 mg *T. maritima *6PGDH/mL, 1 mg BSA/mL, 0.5 mM MnCl_2 _and 5 mM MgCl_2_. A 10 μL of the sample was withdrawn and diluted 20-fold in 5 mM H_2_SO_4_. Xylitol was measured by a HPLC equipped with the Bio-Rad Aminex HPX-87H column [59].

## Competing interests

The authors declare that they have no competing interests.

## Authors' contributions

YW and YHPZ designed the experiment. YW performed the experiments. YW and YHPZ wrote the manuscript. Both authors read and approved the final manuscript.
